# Calcium regulation of muscle spindle mechanosensory afferent function

**DOI:** 10.1113/EP092558

**Published:** 2025-06-27

**Authors:** Anna Simon, Richard A. Lofthouse, Philip Miti, Claudiu V. Giuraniuc, Robert W. Banks, Guy S. Bewick

**Affiliations:** ^1^ School of Medicine, Medical Sciences & Nutrition University of Aberdeen Aberdeen UK; ^2^ Department of Physics University of Aberdeen Aberdeen UK; ^3^ Department of Biosciences University of Durham Durham UK

**Keywords:** calcium‐activated potassium channel, calcium channel, mechanosensation, muscle spindle, proprioception, TRPV4

## Abstract

Extracellular calcium is crucial for the normal function of muscle spindle sensory afferents. They express multiple calcium buffering proteins. Extracellular calcium is essential for recycling of synaptic‐like vesicles (SLVs) in the terminals and for the stretch‐evoked inward calcium current of the receptor potential. Conversely, removal of calcium from the extracellular medium abolishes stretch‐evoked action potentials (APs). However, the calcium channel(s) involved and mechanism(s) of action are unknown. This study begins identifying the channels involved and their actions. Specific calcium channel toxins, agonists and antagonists were examined for effects on stretch‐evoked muscle spindle afferent discharge, and live spindle sensory terminal labelling with FM1‐43 was used to monitor SLV recycling in adult rat lumbrical muscle. Voltage‐gated calcium channels, particularly P/Q‐type (Ca_v_2.1) and L‐type (Ca_v_1.1–1.4), strongly regulated the firing frequency of APs in response to a standard stretch, probably by regulating the opening of ‘big’, ‘intermediate’ and ‘small’ calcium‐activated potassium channels (K_Ca_), with direct evidence for BK (K_Ca_1.1), SK (most likely K_Ca_2.2) and IK (K_Ca_3.1) involvement. Moreover, calcium from two different sources regulated separate aspects of SLV recycling. Thus, L‐type channel blockers inhibited FM1‐43 release, while TRPV4 (transient receptor potential, vanilloid, type 4) channel blockers entirely inhibited FM1‐43 uptake. No role in SLV recycling was found for P/Q type channels, and no role at all was found for N‐type (Ca_v_2.3) channels. Overall, these studies pinpoint multiple different aspects of calcium signalling, through different channel families, and produce the first evidence of a role for a mechanosensory TRPV4 channel in muscle spindle sensory terminal function.

## Abstract (highlights)

- **What is the central question of this study?**
  External calcium is essential for muscle spindle stretch‐evoked nerve firing and sensory nerve terminals express multiple calcium‐buffering proteins, yet calcium hardly contributes to stimulus‐evoked potentials: so what is calcium's role?
- **What is the main finding and its importance?**
  Muscle spindles of *ex vivo* rat muscles revealed multiple roles for calcium. Stretch (TRPV4) and voltage‐activated (L‐type) calcium channels control endo‐ and exocytosis of glutamate, respectively, essential for terminal stretch‐sensitivity. Multiple calcium‐activated potassium channels gated by voltage‐activated (L‐ and P/Q‐type) calcium channels regulate afferent discharge rates encoding muscle length to the CNS.

## 1 INTRODUCTION

There is extensive evidence that calcium is crucial for muscle spindle sensory terminal function. However, the channels and mechanism(s) of action involved are unclear. Here we explore a range of options for what role(s) calcium might play.

The primary endings of mammalian muscle spindles and their Ia axons form the afferent part of the monosynaptic motor reflex, being identified as afferent end‐organs in the 19th century (Ruffini, 1898; Sherrington, 1894). They report skeletal muscle length with high sensitivity to the central nervous system, detecting stimuli through annulospiral terminals wrapped around the central portion of specialised (intrafusal) muscle fibres. The central portion of the muscle spindle is enclosed within a multilayered cellular and fibrous capsule, which provides protection and a special milieu for mechanosensation (Ovalle, 1976). In the primary sensory terminals, the receptor potential in standard extracellular medium depends exclusively on sodium influx (Hunt et al., 1978). However, removing sodium from the extracellular medium reveals the presence of a residual stretch‐activated calcium current. Moreover, muscle spindle primary sensory nerve terminals express at least six different calcium‐buffering proteins (parvalbumin (Celio, 1990), calbindin/D‐28 (Celio, 1990; Hietanen‐Peltola et al., 1992), calretinin (El‐Tarhouni and Banks, 1995), neurocalcin (Iino et al., 1998), NAP‐22 (Iino et al., 2004) and frequenin/NCS‐1 (Werle et al., 2000). These multiple buffers offer different kinetics of calcium buffering (fast, calretinin/calbindin; slow, parvalbumin) and different cytosolic mobilities (high, parvalbumin/calretinin; low, calbindin), indicating intracellular calcium is regulated across a wide range of kinetics and locations (Schwaller et al., 2002).

Although removal of extracellular calcium in the continued presence of sodium has no effect on the receptor potential, action potential (AP) firing is quickly abolished if extracellular calcium is removed or is replaced with cobalt to block calcium channels (Bewick et al., 2005; Kruse & Poppele, 1991). One known role for intracellular calcium is in regulating the exo‐ and endocytosis of synaptic‐like vesicles (SLVs) in the sensory terminals, which secrete glutamate as part of an autogenic modulatory system regulating muscle spindle sensitivity (Bewick et al., 2005; Than et al., 2021). Extracellular calcium is essential for SLV recycling, since cobalt blockade abolishes SLV endocytosis (Bewick et al., 2005). This correlates with the observations that spindle afferent terminals contain much of the machinery for calcium‐regulated vesicular secretion, including synapsin I and synaptophysin (De Camilli et al., 1988), syntaxin IB (t‐SNARE; Aguado et al., 1999) and VAMP/synaptobrevin I and II (v‐SNARE; Li et al., 1996). The identity of the stretch‐activated calcium channel(s) underlying the contribution to the receptor potential or driving SLV recycling is unknown, although L‐type (Ca_v_1.1–1.4) voltage‐gated calcium channels (VGCCs) have been reported to contribute to mechanotransduction and/or the encoding process (Fischer & Schafer, 2002).

VGCCs have three major families: high‐voltage activated (HVA) dihydropyridine‐sensitive (L‐type, Ca_v_1.1–1.3) or ‐insensitive (P/Q type, N‐type and R‐type; Ca_v_2.1–2.3 respectively) channels and low‐voltage‐activated (LVA) (T‐type, Ca_v_3.1) channels (Table 1; https://www.guidetopharmacology.org/). Several HVA channels reportedly regulate mechanosensory function, including Ca_v_1.2 (Lyford et al., 2002; Magra et al., 2007; Peng et al., 2005), Ca_v_1.3 (Cui et al., 2007; Sidi et al., 2004), Ca_v_2.1 (Haeberle et al., 2004) and Ca_v_2.2 (N‐type; Calabrese et al., 2002) channels. Conversely, LVA (Ca_v_3.1–3.3, T‐type) channels seem mainly involved in nociceptive mechanotransduction (Heppenstall & Lewin, 2006; Nowycky et al., 1985). Another common channel family enabling calcium entry into cells are the transient receptor potential (TRP) channels, especially channels of the TRPC (canonical‐type) and TRPV (vanilloid‐type) family, some of which are reported to be mechanically sensitive (Zhang et al., 2023). Finally, the mechanosensory channel Piezo2 is reported as a mechanotransducer in muscle spindle sensory endings (Woo et al., 2015). As, like the TRP channels, it is another non‐selective cation channel, it too could be a source of mechanosensory calcium influx. However, the lack of selective agonists and antagonists, or genetically modified rats, makes it difficult to explore in the *ex vivo* wild‐type tissues used. Thus, Piezo2 involvement has not been investigated here.

**TABLE 1:** Nomenclature of voltage‐gated calcium channels (VGCC), calcium‐activated potassium channels (K_Ca_) and transient receptor potential channels.

	Non‐IUPHAR	IUPHAR	Toxins/ligands used in this study^a^
Voltage‐gated calcium channels	L‐type	Ca_v_1.1–1.4	Nifedipine, TaiCatoxin
	P/Q‐type	Ca_v_2.1	ω‐agatoxin‐IVA (s), ω‐conotoxin‐MVIIC
	N‐type	Ca_v_2.2	ω‐conotoxin‐GVIA (s), ω‐conotoxin‐MVIIC
Calcium‐activated potassium channels	BK	K_Ca_1.1	Iberiotoxin (s), charybdotoxin, NS1619 (s, ag)
	SK1 SK2 SK3	K_Ca_2.1 K_Ca_2.2 K_Ca_2.3	Apamin, TaiCatoxin
	IK	K_Ca_3.1	TRAM‐34 (s), charybdotoxin
Transient receptor potential channels		All TRPs	2‐APB
		TRPV1	Capsaicin (s, ag)
		TRPV2	Tranilast (s), 2‐APB (ag)
		TRPV3	Carvacrol (s, ag)
		TRPV4	RN1734 (s), GSK1016790A (s, ag)

^a^Blockers/antagonists unless otherwise stated. s, selective; ag, agonist. From https://www.guidetopharmacology.org/

In addition to SLV recycling, other targets for intracellular calcium are calcium‐activated potassium channels. There are at least two potassium permeabilities contributing to the sensory terminal receptor potential. One is sensitive and the other insensitive to tetraethylammonium (TEA) (Hunt et al., 1978). VGCCs are commonly co‐expressed with calcium‐activated potassium channels (K_Ca_s), which are less sensitive to TEA than voltage‐gated potassium channels. As a partnership, VGCCs and K_Ca_s commonly regulate neuronal excitability both in the CNS (Berkefeld et al., 2006; Edgerton & Reinhart, 2003; Marrion & Tavalin, 1998; Womack et al., 2004) and in peripheral mechanosensitive cell types, including neurons (Holm et al., 2000; Kruse & Poppele, 1991; Piskorowski et al., 2008). K_Ca_s form five well‐defined phylogenetic groups (Wei et al., 2005). The first is the K_Ca_1.1/KCNMA1, or ‘big conductance’ (BK) channel, activated by both voltage and internal calcium. The second is the ‘intermediate‐conductance’ (IK) (K_Ca_3.1, KCCN4) channel. The third is the family of voltage‐insensitive, internal calcium‐activated ‘small conductance’ (SK) (K_Ca_2.1–2.3, KCCN1‐3) channels. The final two groups (K_Ca_4.1, 4.2/KCNMT1‐2, and K_Ca_5.1/KCNMU1) are more sensitive to other intracellular ligands, such as sodium (Yuan et al., 2003). These channels all increase membrane permeability to potassium, driving the membrane potential back towards the potassium Nernst potential, that is, hyperpolarisation (reviewed by Vergara et al., 1998). We have shown that certain K_Ca_s, specifically SK2 (K_Ca_2.2), are expressed in muscle spindle afferent endings, where they are expressed in the terminal and the preterminal axon. We have also modelled their putative role in muscle spindle afferent AP generation and discharge frequency (Housley et al., 2024). However, precisely which calcium channels are present, whether there are other K_Ca_s present in mechanosensory nerve terminals and what roles they all might play are still unclear. We therefore tested for the presence of a number of candidate channels and their functions in the mechanosensory terminals of the muscle spindle.

Here we present physiological and pharmacological evidence for several different major roles for calcium in muscle spindles, which would correlate with the need for extensive calcium buffering. These include Ca_v_2.1 (P/Q‐type) interacting with K_Ca_1.1 (BK), K_Ca_2.1–2.3 (SK) and K_Ca_3.1 (IK) channels to strongly regulate firing, more modest involvement of Ca_v_1.1–1.4 (L‐type) and K_Ca_2.1–2.3 (SK) as modulators of afferent firing frequency in the muscle spindle, plus TRPV4 expressed on the terminals, where it is essential for stretch‐evoked firing, and together with Ca_v_1.1–1.4 (L‐type) regulates SLV exocytosis, but not endocytosis.

## 2 METHODS

### 2.1 Ethical approval

Experiments were performed in compliance with *Experimental Physiology*’s policies. Animals were kept in accordance with UK Home Office standards and ARRIVE guidelines, and as approved by the University of Aberdeen Animal Welfare and Ethical Research Board. Rats were the Sprague–Dawley strain, bred in‐house and kept in a 12 h light–12 h dark regime (7 am on, 7 pm off), at temperatures between 20 and 24°C with ad libitum access to food and water, eating standard rat chow. Housing was in groups wherever possible, in large cages (H 88 cm × W 94 cm × D 65 cm) with a maximum of eight to a cage. All experiments used *ex vivo* tissue obtained after humane killing by the approved strict standards set out in Animals (Scientific Procedures) Act 1986, Schedule 1 in the UK and in Annex IV in the European Directive 2010/63/EU, specifically by placing them in a chamber and exposing them to rising CO_2_ concentrations. Death was ascertained by both verification that the heart beat had stopped and by cervical dislocation.

### 2.2 Animals and dissection

Adult Sprague–Dawley rats (75 male for electrophysiology, 4 female for FM1‐43 labelling; 250–480 g) were used. Specific numbers for each experimental group are given when each result is presented. Fourth lumbrical nerve–muscle preparations from both hind paws and saphenous nerves from the hind legs were dissected, cleaned and mounted in silicone rubber‐lined (Sylgard, Dow Corning, Stade, Germany) culture dishes under constantly carbogenated (95% O_2_–5% CO_2_) saline containing (mM): 138.8 NaCl, 4 KCl, 12 NaHCO_3_, 1 KH_2_PO_4_, 1 MgCl_2_ and 11 glucose (Liley, 1956), pH 7.0–7.2. All experiments were performed at room temperature (19–21°C).

### 2.3 Drug preparation

Final concentrations of VGCC blockers ω‐agatoxin‐IVA (P/Q‐type; Sigma‐Aldrich, Gillingham, UK), ω‐conotoxin‐MVIIC (N‐ and P‐type), and ω‐conotoxin‐GVIA (N‐type; Peptide Institute Inc., Osaka, Japan), K_Ca_ blockers charybdotoxin (BK and IK), iberiotoxin (BK, Alomone Laboratories, Jerusalem, Israel) and apamin (SK1‐3, Sigma‐Aldrich), solutions were prepared with Liley's saline each day from frozen stock in the same saline. TRAM‐34 (IK inhibitor, Tocris, Bristol, UK), NS1619 (BK channel activator; Sigma‐Aldrich) and TRP channel ligand (2‐aminoethoxydiphenylborane (2‐APB), tranilast, carvacrol, RN1734 and GSK1016790A, all Tocris) stock solutions were made in dimethylsulfoxide (DMSO). Nifedipine stock (L‐type channel inhibitor; Sigma‐Aldrich) was made up in either ethanol or DMSO. Fresh aliquots of each drug were defrosted each day, then diluted to the final concentration with gassed physiological saline and used immediately. Experiments with the photosensitive drugs NS1619 and nifedipine were kept in the dark between recording sessions and light exposure was minimised throughout.

### 2.4 Electrophysiology

Nerve–muscle preparations were maintained and electrophysiological recordings of stretch‐evoked responses were performed as detailed previously (Bewick et al., 2005; Simon et al., 2010). The fourth lumbrical muscle contains no Golgi tendon organs, so the recorded discharge was only from spindle afferents (Banks et al., 2009). Signals were amplified (A103, Isleworth Electronics, London, UK and 8102, C.F. Palmer, Harvard Instruments, Holliston, MA, USA preamplifiers in series; or Neurolog NL104 and NL106, Digitimer, Welwyn Garden City, UK, in series), displayed on an oscilloscope (DSO 400, Gould Electronics, Freiburg, Germany) and captured on computer hard‐drive (WinWCP software, John Dempster, University of Strathclyde, UK). Stretch–hold–release (1 mm, ∼10% muscle length) cycles and electroneurogram recording were performed as described earlier (Bewick et al., 2005; Lin et al., 2016). Ramp–hold–release stretch stimuli, in groups of four, were applied each 15 min (typically <6 full cycles) until three consistent electroneurograms were recorded. The final recording of these three was used as the predrug control firing level, and then the drug was applied. The reversibility of drug actions was established by washing for at least 1 h in drug‐free saline. Parallel no‐drug control preparations were made with the same protocol and over the same time course.

The purely sensory composition of the saphenous nerve and its great length made it a suitable subject for compound action potential (CAP) measurements, which tested the drugs for nerve conduction block. While it does not contain muscle spindle afferents, it is a preparation suitable for testing for generalised effects on sensory neuron AP amplitude and conduction. The maximum available length of the rat saphenous nerve was excised and placed under carbogenated Liley's saline. Stimulation was applied at one end with a suction electrode, then the CAP was recorded *en passant* with Ag‐wire electrodes at the other extremity. The CAP amplitude was measured as described previously (Simon et al., 2010). Groups of 12 CAPs were recorded every 5 min. Again, time matched control preparations were run in parallel.

### 2.5 FM1‐43 labelling

Sensory terminal labelling of rat lumbrical muscle spindles with FM1‐43, to study SLV endo‐ and exocytosis, was performed as previously described (Bewick et al., 2005). Briefly, muscles were immersed in 10 µM FM1‐43 in Liley's saline, made from a 1 mM freshly defrosted stock, for 2 h at full extension of the muscle, to maximise SLV recycling. Muscles were gassed (95% O_2_–5% CO_2_) continuously. Every 30 min, muscles were briefly returned to minimum unloaded length (×4) before returning to full extension. To ensure blocker penetration before dye application, muscles were preincubated (30 min) in drug/toxin prior to dye incubation with the same drug/toxin. DMSO at the requisite level (always <0.01%) or ethanol served as vehicle controls where relevant. Muscles were returned to unloaded length, and bulk dye was removed by 3× rinses in dye‐ and drug‐free Liley's saline. Then, they were placed in gassed Liley's saline containing 1 mM Advasep‐7, to chelate the dye (∼20 min). Tendons and any final excess connective tissue were removed, then muscles placed on slides in saline, covered with a coverslip and gentle pressure applied (10 mL glass bijou of water) to flatten and maximise visibility. Images were taken at ×20 magnification (Nikon Optiphot II, or Nikon Eclipse Ti, Nikon Instruments, Surbiton, UK).

### 2.6 Data analysis

Experiments and data analysis were not undertaken under blinded conditions.

#### 2.6.1 Electrophysiology

**FIGURE 1:**
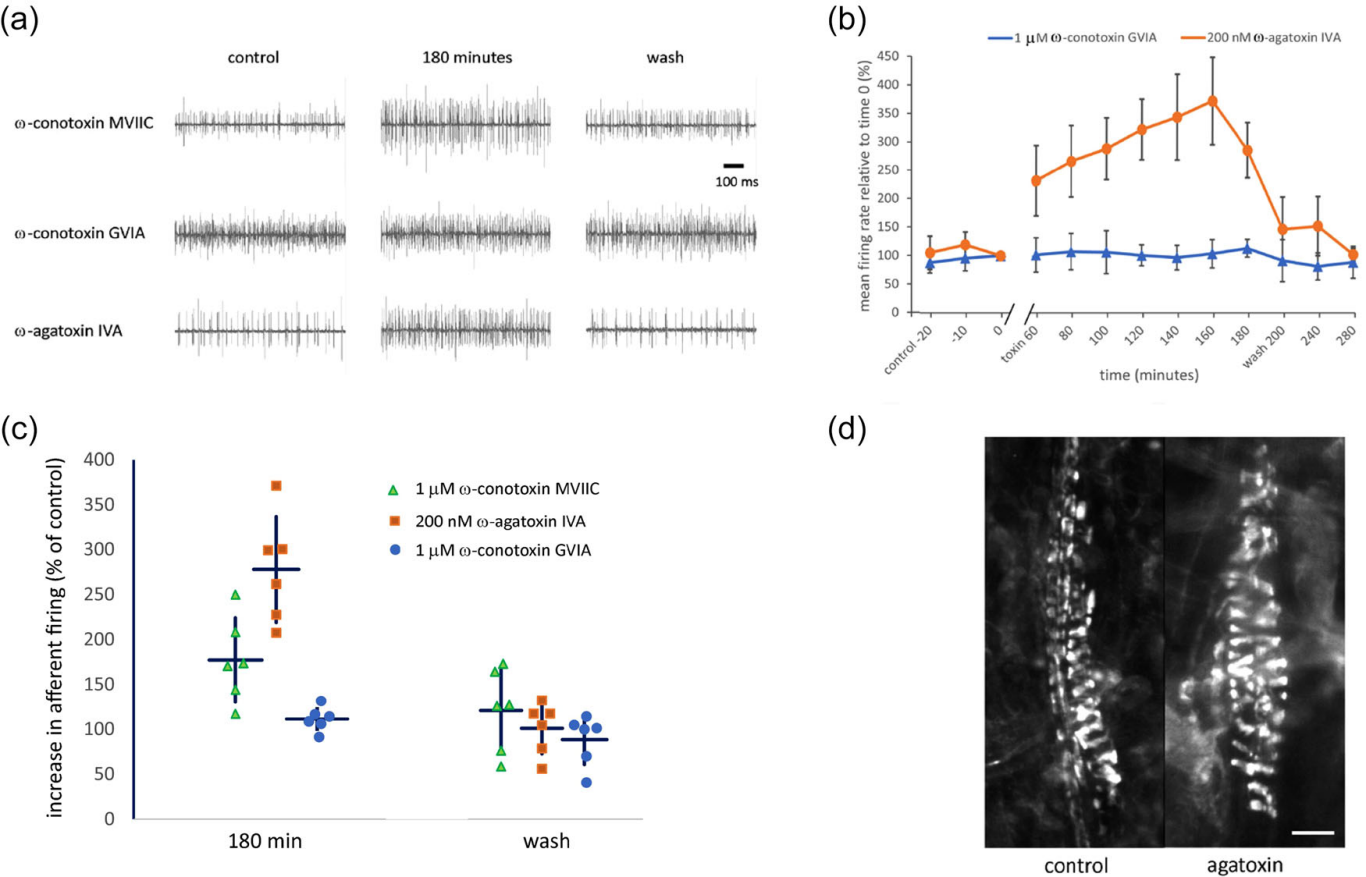
Ca_v_2.1 (P/Q‐type), but not Ca_v_2.2 (N‐type), channels regulate stretch‐evoked spindle afferent firing. Electroneurograms of hold‐phase spindle firing during 1 mm stretches of rat 4th lumbrical muscles for predrug control, in the presence of VGCC blockers and after wash. (a) The N‐ and P/Q‐type VGCC blocker ω‐conotoxin‐MVIIC (1 µM) and the P/Q‐type VGCC blocker ω‐agatoxin‐IVA (200 nM) significantly increased the firing frequency, but the selective N‐type VGCC blocker ω‐conotoxin‐GVIA (1 µM) had no effect. (b) Mean data comparison of the time course of the effects on firing of selective N‐ (*n* = 6) and P/Q‐type VGCC blockade (*n* = 6). (c) Mean data comparison of the maximal firing for the three toxins: ω‐conotoxin‐MVIIC (1 µM), which blocks both N‐ and P‐type calcium channels (*n* = 5; *P *= 0.002), ω‐agatoxin‐IVA (200 nM), which blocks P/Q‐type channels and increased firing most (*n* = 6; *P *< 0.001), and ω‐conotoxin‐GVIA (1 µM), which completely and irreversibly blocks N‐type VGCCs but had no significant effect on afferent firing (*n* = 6; *P *= 0.629). (d) ω‐Agatoxin‐IVA (200 nM), the most potent toxin on hold‐phase firing, had no effect on FM1‐43 dye uptake labelling intensity. Scale bar = 20 µm.

**FIGURE 2:**
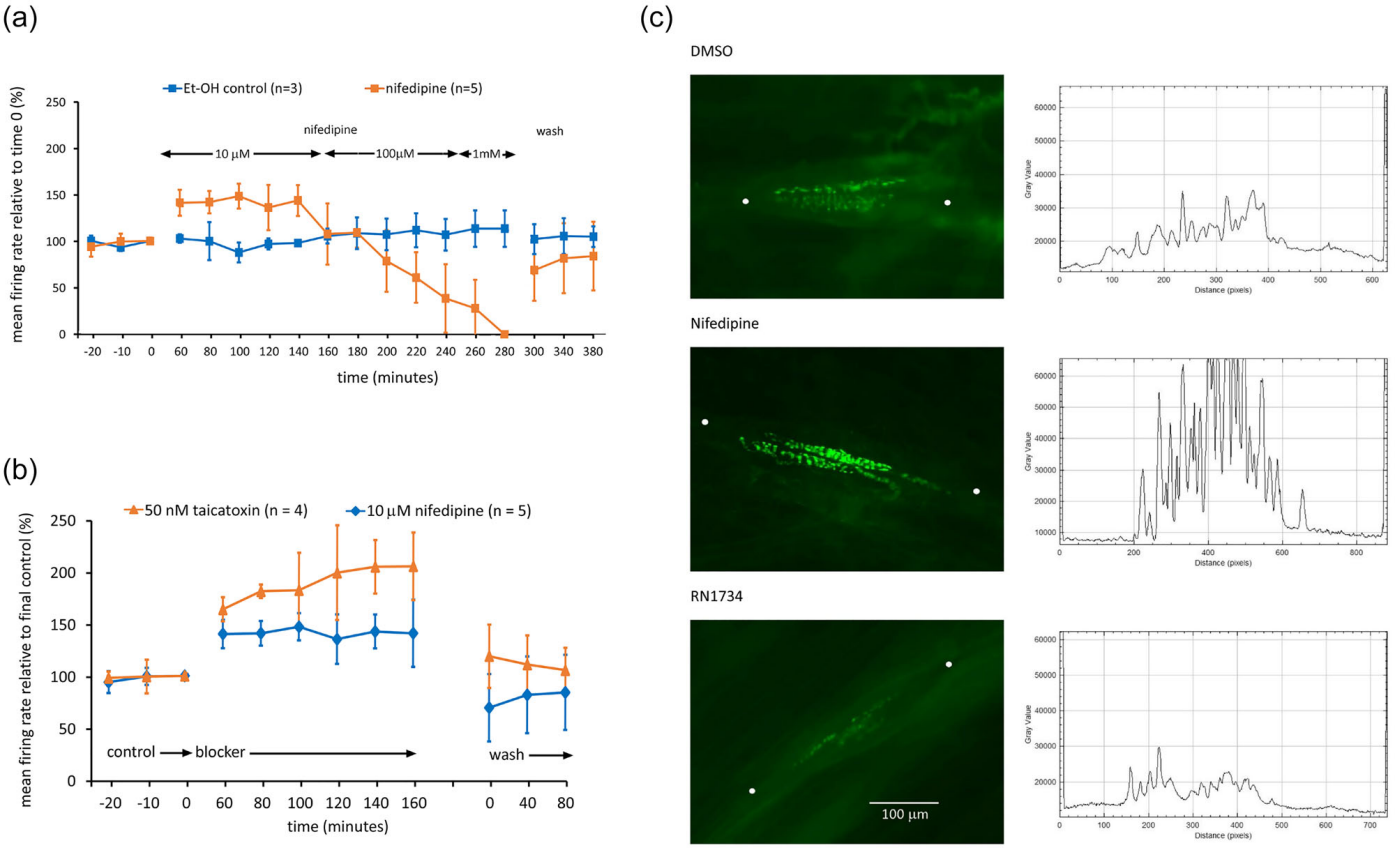
Ca_v_1.1–1.4 (L‐type) channels regulate stretch‐evoked spindle afferent firing and SLV recycling. (a). Nifedipine (L‐type blocker, *n* = 5) initially enhanced firing (10 µM, *P *= 0.001), but then totally inhibited at higher concentrations (*P *< 0.001). (b). Taicatoxin (50 nM, L‐type and SK/K_Ca_2.1–2.3 blocker, *n* = 4) enhanced firing even more than 10 µM nifedipine (*P *< 0.001). (c) Compared to DMSO controls (29 spindles), nifedipine (L‐type channel blocker, 26 spindles, 3 rats) greatly increased FM1‐43 retention by sensory nerve terminals (*P *< 0.001) while RN1734 (TRPV4 antagonist, 32 spindles, 3 rats) substantially decreased FM1‐43 labelling (*P *= 0.032). Right column, intensity profile plots along a straight line between the two white dots. All data are from 1–3 muscles per rat.

**FIGURE 3:**
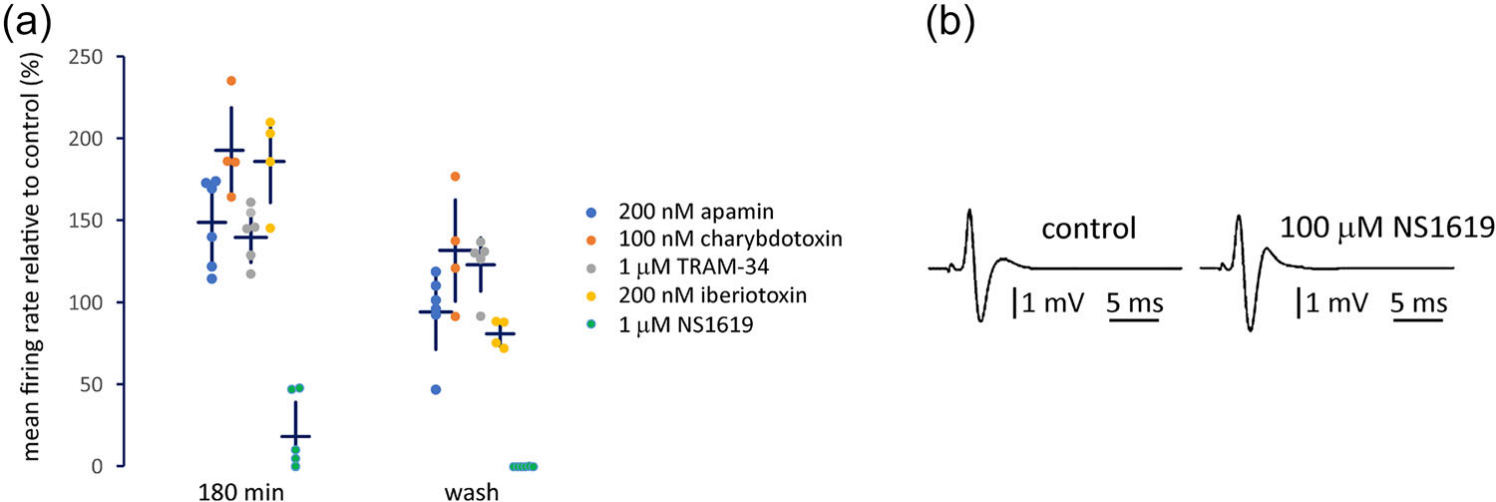
K_Ca_ inhibition enhances firing. (a) BK (iberiotoxin and charybdotoxin), IK (TRAM 34 and charybdotoxin) and SK (apamin) inhibitors increased stretch‐evoked firing compared to their predrug control (= 100%) (iberiotoxin, *n* = 4, *P *< 0.001; charybdotoxin, *n* = 4, *P *= 0.017, TRAM 34, *n* = 5, *P *= 0.001; apamin, *n* = 6, *P *= 0.003). Conversely, BK activation (NS1619, 1 µM) entirely abolishes firing in most muscles (*n* = 6, *P *< 0.001). (b). NS1619, even at 100 µM, had no effect on propagation or amplitude of the CAP in saphenous nerve of the same animals.

**FIGURE 4:**
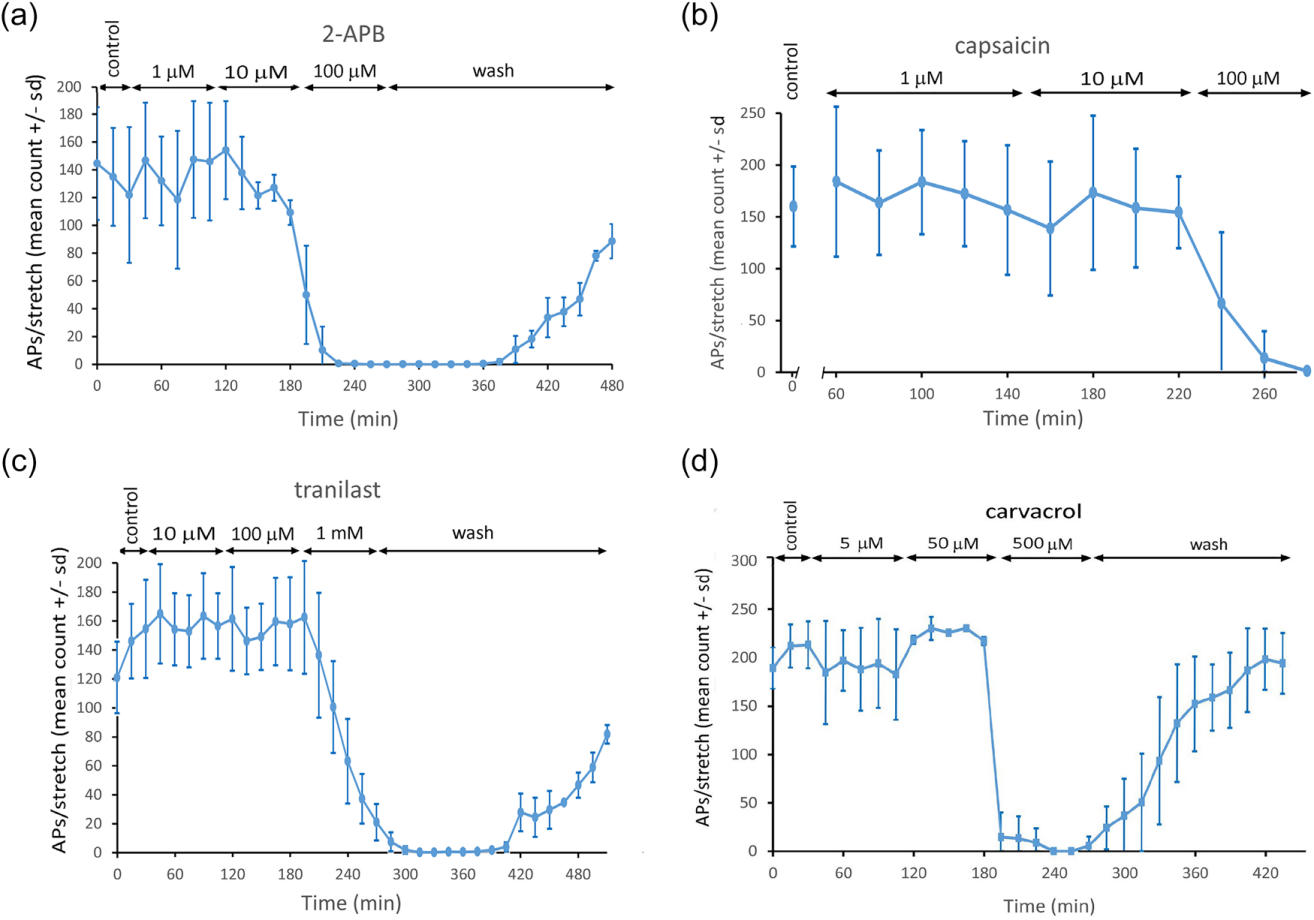
Broad‐spectrum TRP antagonist and TRPV1–V3 ligands block stretch‐evoked firing. (a) 2‐APB, a broad spectrum TRP inhibitor, blocked stretch‐evoked firing at 100 µM (*n* = 3, *P *= 0.049). (b) Capsaicin, a TRPV1‐selective agonist at 300 nM (*n* = 6), had no effect up to 10 µM (*P *= 0.792), although it did block at very high concentrations (100 µM, *P *< 0.001). (c) Tranilast, a selective TRPV2 antagonist up to 10 µM (*n* = 3), had little effect up to 100 µM but blocked firing completely at 1 mM (*P *= 0.009). This is at 10× above the selective concentration range, suggesting inhibition is an off‐target effect. (d) Carvacrol, a TRPV3, TRPA1 and K2P2.1 agonist (*n* = 3), blocked stretch‐evoked firing at 500 µM (*P *= 0.004), which is its normal active range for action on TRPV3. However, blocking firing with TRP channel agonism is hard to interpret and suggests this is either due to K2P2.1 activity or an off‐target effect.

**FIGURE 5:**
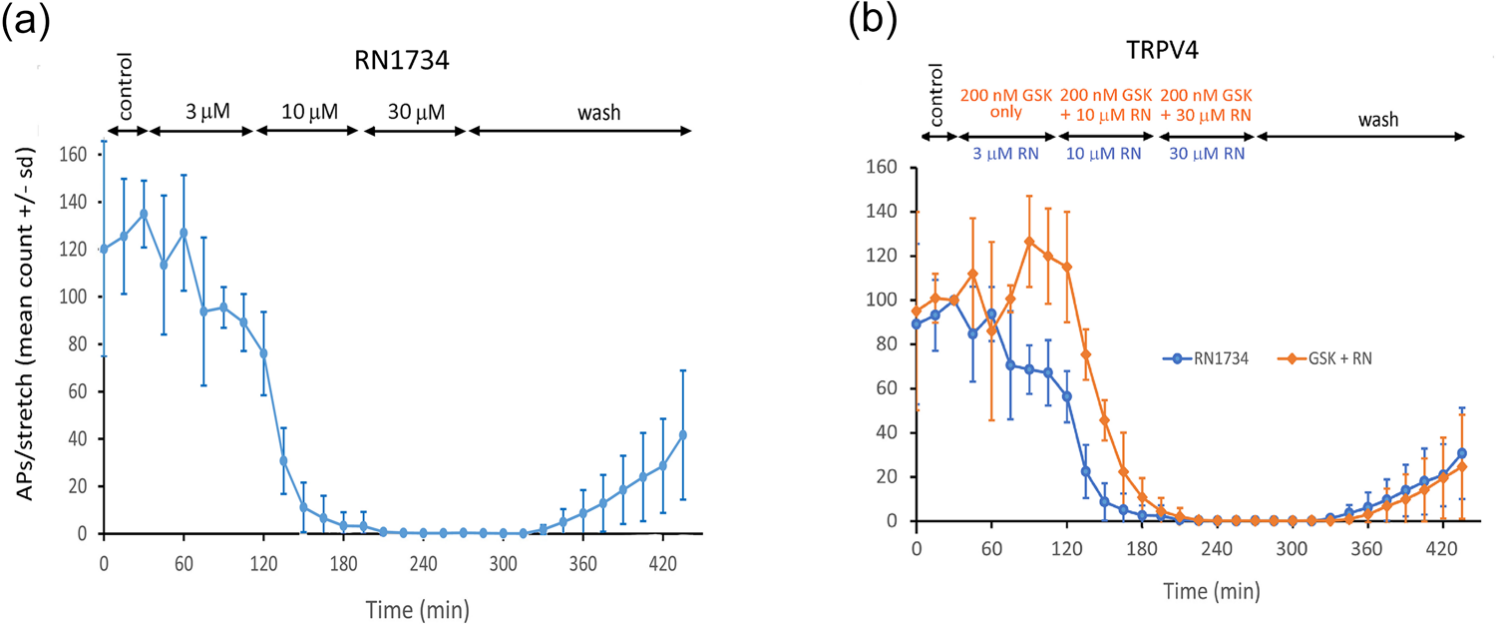
TRPV4 channel ligands modulate stretch‐evoked firing. (a) RN1734, a selective TRPV4 antagonist (IC_50_ 6 µM), inhibited hold‐phase firing at 3 µM (*n* = 4, *P *= 0.034) and blocked it completely at 30 µM (*P *< 0.001). (b) GSK101‐7690, a potent TRPV4 agonist (18 nM; *n* = 5), at 200 nM slightly increased hold‐phase firing (*P *= 0.036) and reduced the rate of onset of inhibition by 10 µM RN1734. These data indicate that TRPV4 is present and has powerful effects on stretch‐evoked firing.

For Figures 1, 2, 3, stretch stimulus application and mean firing count for the first second of the hold phase (APs per second) were performed manually, while CAP amplitude was analysed, as previously reported (Bewick et al., 2005; Lin et al., 2016; Simon et al., 2010). For Figures 4 and 5 stretch stimulus and counting were both automated, so counts for the entire hold phase are reported (either as per Lin et al., 2016, or MATLAB codes developed for this analysis. MATLAB codes and full instructions are freely available at: https://github.com/cgiuraniuc/Spindle-Firing-Analysis.git). [Correction made on 27th August 2025, after first online publication: Electrophysiology section has been updated.]. Only static output was reported because stretch was applied manually for Figures 1, 2, 3 via a micromanipulator with a vernier scale (MM3, Narishige, UK). Dynamic phase responses were therefore likely affected by inherent variability of elongation rates in manually stretched preparations, making accurate quantification and outcome interpretation impossible. Data are expressed as means ± SD and differences between means were evaluated by paired Student's *t*‐test unless otherwise specified, with a significance threshold of *P* = 0.05.

#### 2.6.2 Fluorescence intensity analysis

Images were obtained from three (DMSO), two (nifedipine) and three (RN1734) muscles from hindfoot lumbrical muscles of four adult female (350–470 g) Sprague–Dawley rats. They were imported into Photoshop CS2 (Adobe, San Jose, CA, USA), to enable a pair of maximal intensity white marker dots to be added for subsequent line‐scan analysis using Fiji (https://imagej.net/software/fiji/); examples are shown in Figure 2c. Data from the line scans were imported into Microsoft Excel for construction of intensity histograms, each of 20 bins. All line scans were dominated by low‐intensity pixels, so the proportion of the total number of pixels in the 15 highest intensity bins to the overall total of pixels in all 20 bins was calculated for comparison. Totals of 29 (DMSO treated), 26 (nifedipine treated) and 32 (RN1734 treated) spindles were analysed in this way.

## 3 RESULTS

### 3.1 Voltage‐gated Ca^2+^ channel regulation of afferent discharge

Given that sensory terminals rely on the release of glutamate from SLVs to maintain their stretch‐sensitivity, and that vesicle recycling at synapses typically is regulated by VGCCs, we first investigated the importance of the HVA Ca^2+^ channels – P/Q‐type (Ca_v_2.1), N‐type (Ca_v_2.2), and L‐type (Ca_v_1.1–1.4) for stretch‐evoked, hold‐phase, firing (Figure 1).

### 3.2 N‐ and P/Q‐type VGCC blocker increases spindle afferent firing

To determine if Ca_v_2.1 (P/Q‐type) or Ca_v_2.2 (N‐type) VGCCs play an important role in spindle mechanotransduction, we tested the effect of blocking both, with ω‐conotoxin‐MVIIC (Wheeler et al., 1994). Somewhat surprisingly, the toxin (1 µM) doubled the stretch‐evoked afferent firing (*n* = 5; *P *= 0.002. Figure 1). Enhanced hold‐phase firing was maintained throughout the 3‐h incubation and reversed entirely on washing (*P *= 0.675). Thus, at least one of these channel types is present and modulates firing. We next investigated which of the two VGCCs, N‐ or P/Q‐type, was involved.

### 3.3 N‐type VGCC blocker has no effect on spindle afferent firing

To determine if it is the Ca_v_2.2 (N‐type) channel blockade that enhances firing, ω‐conotoxin‐GVIA was applied, a specific, high affinity irreversible blocker of these channels (Cruz & Olivera, 1986; McCleskey et al., 1987). Although the toxin blocks in the mid‐nanomolar range (Ellinor et al., 1994), even 1 µM had no effect on stretch‐evoked spindle afferent firing (Figure 1, *n* = 6; *P *= 0.629) indicating Ca_v_2.2 VGCCs were not the cause of the increase in stretch‐evoked muscle spindle firing.

### 3.4 Selective P/Q‐type VGCC blockers greatly increase afferent firing

We therefore next tested directly for the role of Ca_v_2.1 (P/Q) channels, by applying the selective inhibitor ω‐agatoxin‐IVA (Luebke et al., 1993) (Figure 1; Olivera et al., 1994). After 80 min, 200 nM ω‐agatoxin‐IVA essentially doubled the hold‐phase afferent nerve firing to a similar extent to ω‐conotoxin‐MVIIC (*n* = 6; *P *< 0.001). Hold‐phase firing then continued to increase with continued application, with stretch‐evoked firing reaching 325.3 ± 92.6% of control after 2 h (*P *< 0.001). The increase in hold‐phase firing was entirely reversible with a 1‐h wash (*P *= 0.291; Figure 1). Thus, it appears that it is the Ca_v_2.1 (P/Q) channels that were regulating afferent discharge.

### 3.5 P/Q‐type voltage‐gated Ca^2+^ channels regulate firing but not SLV recycling

One possible reason for increased stretch‐evoked firing with Ca_v_2.1 blockade is if the toxin were to increase SLV recycling, and hence release of glutamate, from spindle sensory terminals. Since we have shown previously that SLVs take up and release FM1‐43 in a reversible and calcium‐dependent manner (Bewick et al., 2005), we tested if ω‐agatoxin‐IVA, the specific P/Q‐type VGCC toxin, blocked vesicular uptake of FM1‐43 into rat muscle spindle sensory terminals. Even at very high concentrations (200 nM), ω‐agatoxin‐IVA had no effect on FM1‐43 uptake (Figure 1d). Thus, the increased firing is not due to increased SLV recycling. Rather, this VGCC seems to directly regulate afferent firing.

### 3.6 L‐type VGCC blocker has a biphasic effect on firing

Uncovering a role for Ca_v_2.1 does not, of course, preclude a role for other types of VGCCs, so evidence for the other HVA channel was examined. To test for a role for L‐type VGCCs (Ca_v_1.1–1.4), we applied progressively increasing concentrations (0.01–1 mM) of nifedipine. Nifedipine at 10 µM increased firing by around an additional 40% above control (*n* = 5, *P *= 0.001) soon after application, an effect which was maintained (Figure 2a). At this concentration, it is a selective L‐type channel blocker (Fischer & Schafer, 2002). At higher concentrations, where it would begin to have substantive off‐target effects, it progressively reduced stretch‐evoked firing. Thus, 100 µM decreased the afferent firing down to 38.8 ± 36.4% of control (*P* = 0.028), while at 1 mM, nifedipine blocked stretch‐evoked afferent discharge completely in four out of five experiments (*P *< 0.001; Figure 2a). At this concentration, nifedipine often also induced spontaneous muscle twitching, presumably reflecting effects on the extrafusal skeletal muscle dihydropyridine receptors, which are also L‐type channels, or blockade of voltage‐gated potassium channels. These effects were all reversible upon wash. Thus, blocking L‐type channels with low concentrations of nifedipine also produced excitation, as observed with P/Q‐type‐blockers, while higher concentrations inhibited, indicating additional actions on other targets.

### 3.7 Taicatoxin (L‐type and SK2 blocker) increases stretch‐evoked firing

Given these mixed effects with nifedipine, as another test for the involvement of L‐type VGCC, taicatoxin was applied. This blocks L‐type VGCCs (Possani et al., 1992) but possibly also SK2 K_Ca_ channels (Doorty et al., 1997). From our results above, blocking either (or both) of these channels would be expected to increase afferent discharge. Consistent with this, 50 nM taicatoxin significantly enhanced afferent firing during a 1‐h incubation. Stretch‐evoked firing frequency continued to increase for the remainder of the 3‐h incubation, reaching 230.0 ± 28.6% (*n* = 4; *P *= 003; Figure 2b). Higher concentrations (300 nM) had no additional effects, again doubling the firing frequency by the end of the 3‐h incubation period (*n* = 5; *P *= 0.001). These observations are largely consistent with previous selective VGCC blockade outcomes, indicating VGCCs regulate firing.

### 3.8 L‐type calcium channels regulate SLV recycling

To determine if L‐type channels might be a source of calcium to modulate SLV recycling, we examined the effects of low concentration nifedipine (10 µM) on FM1‐43 internalisation (Figure 2c). Somewhat counter to expectations, nifedipine greatly increased the FM1‐43 labelling of annulospiral endings. Mean intensity proportions were: DMSO treated, 0.135 ± 0.182; nifedipine treated, 0.376 ± 0.230 (*t*‐test, *P* < 0.001). Clearly, blocking L‐type VGCCs did not inhibit dye uptake; rather, it was enhanced.

Overall, therefore, L‐ and P/Q‐type VGCCs negatively regulate firing, with P/Q‐type being the most powerful. Conversely, L‐type VGCCs, but not P/Q‐type channels, regulate at least some aspects of SLV recycling (FM1‐43 uptake). The enhanced firing implies calcium might be opening K_Ca_s. We therefore next tested for such a functional interaction.

### 3.9 K_Ca_ channel blockers increase, and activators decrease, afferent firing

VGCCs are commonly co‐expressed with K_Ca_s, as a regulatory mechanism for neuronal excitability, particularly P/Q‐type VGCCs (Berkefeld et al., 2006; Edgerton & Reinhart, 2003; Marrion & Tavalin, 1998; Womack et al., 2004). Also, we have shown K_Ca_2.2 (SK2) is expressed in the preterminal axon and terminals (Shenton et al., 2014). We therefore tested for a functional role for K_Ca_ channels.

If firing regulation involves K_Ca_s, then blocking these channels directly should have the same effect as ω‐conotoxin‐MVIIC and ω‐agatoxin‐IVA. Consistent with this (Figure 3a), apamin (200 nM), an inhibitor of all small conductance (SK, K_Ca_2.1–2.3) channels, increased afferent firing to 50% above control (*n* = 6, *P* = 0.003). In addition, charybdotoxin (100 nM), a blocker of K_Ca_1.1 (BK) and K_Ca_3.1 (IK) channels, almost doubled stretch‐evoked firing (*n* = 4; *P *= 0.019).

To differentiate between BK and IK channels, the effects of TRAM 34, a selective K_Ca_3.1 (IK) blocker, and iberiotoxin, a blocker of K_Ca_1.1 (BK), were compared. TRAM 34 at 1 µM increased afferent firing by 40% above predrug control values at 3 h incubation (*n* = 5, *P *= 0.001), but iberiotoxin (200 nM), from the Indian red scorpion (*Hottentotta tamulus*, formerly *Buthus tamulus*; Candia et al., 1992), had the greatest effect, more than doubling initial firing at this time (*n* = 4, *P* < 0.001). Higher concentrations of these blockers had no additional effect, indicating these were maximal effects for each drug. Interestingly, all enhancements progressively increased throughout the 3‐h incubation time. Despite this, even after 3 h, in most cases spindle activity returned to the control level during the standard 1‐h wash. The only exception was TRAM 34, where there was no significant decrease in the firing frequency after even 90 min wash (*P *= 0.251), consistent with its action being only slowly reversible (Stoneking, et al., 2013).

These data indicate that members of all three channel families (BK, IK and SK) are functionally important. As an additional test of this model we asked if an agonist of these channels decreased firing. NS1619, which is a specific and irreversible activator of K_Ca_1.1 (BK) channels, had the strongest effect on firing. NS1619 at 1 µM produced a profound decrease of stretch‐evoked afferent firing (*n* = 6, *P *= 0.001) after 3 h of incubation (Figure 3a). The effect was again slowly progressive, such that a total block often only occurred during the initial wash phase and the abolition was not reversible during the standard 1‐h wash. This reduction in stretch‐evoked firing was not due to NS1619 directly inhibiting AP propagation, since even 100 µM (100×) concentration had no effect on the CAP nerve conduction, amplitude or shape in the sensory saphenous nerve (*n* = 6) over 80 min (Figure 3b).

Thus, overall, these observations indicate VGCC blockade, at least initially, consistently increased stretch‐evoked firing. The possible mechanisms for the later inhibition due to blocking L‐type channels by nifedipine will be considered in the Discussion. Thus, in general, inhibiting VGCCs does not explain the blockade of firing in the absence of calcium. We therefore investigated whether blocking another, more likely mechanosensitive, Ca‐permeant channel candidate family might inhibit firing. These were the TRP channels, for which a range of selective pharmacological tools exist.

### 3.10 TRPV4 channel inhibition inhibits stretch‐evoked afferent firing

TRPs are non‐selective cation channels, and due to a significant permeability to calcium, are often involved in calcium signalling (Zhang et al., 2023). To test for any TRPs functionally relevant to stretch‐evoked firing, the non‐selective blocker 2‐aminoethoxydiphenylborate (2‐APB) was applied (Bootman et al., 2002). 2‐APB acts on all TRPs, primarily as an inhibitor, but it activates TRPV1–3. 2‐APB at 1 and 10 µM had little effect on stretch‐evoked firing but at 100 µM it completely blocked responses (*n* = 3, *P *= 0.0497), an effect which was slowly reversible (Figure 4a). Of all the TRP channels, TRPV4 has the most evidence for being mechanically sensitive (O'Neil & Heller, 2005). Experiments therefore next focussed on testing for evidence of TRPV channel involvement. TRPV1–3 ligands all had effects on firing, mostly inhibitory, but only at very high concentrations above their selectivity range. The potent TRPV1 agonist capsaicin (selective in the 100–300 nM range; Bevan & Szolcsányi, 1990), had no significant effect on hold‐phase firing until 100 µM, whereupon it blocked entirely (*n* = 6). We have shown previously that capsazepine, a selective TRPV1 antagonist up to 10 µM, had little effect on firing even at 100 µM (Simon et al., 2010). The TRPV2 antagonist tranilast (selective at 50–200 µM; Iwata et al., 2018; Nie et al., 1997) only reduced firing at 1 mM (Figure 4c), when firing ceased entirely (*n* = 3, *P *= 0.009). This latter blockade had a very slow onset, reaching total abolition 2 h after exposure, which was 1 h after washout of the drug began. Thus, drugs testing for TRPV1 and TRPV2 involvement only had actions well above their range of specificity, suggesting any inhibition was likely due to off‐target effects. Carvacrol, an agonist at TRPV3 (at 500 µM), TRPA1 (at 200 µM) and K2P2.1 (TREK‐1, at 300 µM) (Mukaiyama et al., 2020; Xu et al., 2006), had no effect at 50 µM, but rapidly, profoundly and reversibly inhibited firing at 500 µM (*n* = 3, *P *= 0.004; Figure 4d), indicating the possible presence of one or more of these three channel subtypes. While it is unclear whether TRPV3 is present, the activation‐induced inhibition of firing suggests blocking calcium entry through these channels does not explain the profound inhibition seen in zero calcium saline (Kruse and Poppele, 1991).

The final TRP receptor investigated was TRPV4, a well‐established candidate mechanosensory channel (Gualdani et al., 2020). In contrast to the high concentrations of action for the other TRPVs, RN1734, a selective TRPV4 inhibitor (IC_50_ 6 µM; Vincent et al., 2009) significantly inhibited hold‐phase firing at 3 µM (*n* = 4, *P *= 0.034), and completely abolished hold‐phase firing at 10 µM (*P *< 0.001; Figure 5a). This recovered substantially with 2.5 h of wash. GSK1017690A, a potent selective TRPV4 agonist (EC_50_ 18 nM; Thorneloe et al., 2008) at 200 nM modestly increased stretch‐evoked firing (*n* = 5, *P *= 0.036; Figure 5b) after 1 h incubation. Further, it did reduce the rate of inhibition by 10 µM RN1734.

The potency and specificity of these TRPV4 ligands, together with the substantive responses to them, indicate TRPV4 is a strong candidate stretch‐sensitive calcium channel in muscle spindles.

### 3.11 TRPV4 regulates SLV recycling

Since calcium‐dependent release of glutamate enhances firing, we next examined if TRPV4 might regulate SLV recycling. RN1734 substantially decreased FM1‐43 internalisation (Figure 2c; RN1734 treated, 0.051 ± 0.092 SD, *n* = 32 spindles; DMSO treated, 0.135 ± 0.182 SD, *n* = 29 spindles; *t*‐test, *P* = 0.032). This implies calcium entry via TRPV4 channels regulates FM1‐43 fluxes but a different aspect than L‐type calcium channels.

## 4 DISCUSSION

Given the powerful effects of extracellular calcium removal, and the substantial number of calcium buffering proteins within the sensory terminal, this study investigated the role(s) of calcium in muscle spindle sensory terminal function. While the immunofluorescence, immunohistochemical and FM1‐43 uptake studies here and previously give very precise localisation of the various calcium buffering proteins, K_Ca_s and sites of FM1‐43 recycling when associated with the mechanosensory terminals, it is also important to consider that functional effects of channel pharmacology may not be confined to the sensory terminals. This is because muscle spindle outputs are the end‐product of a complex interaction between the intrafusal muscle fibres and the sensory terminals, plus modulation of the encoding site(s), thought to be the first heminode of Ranvier after the terminal (Carrasco et al., 2017; Quick et al., 1980). Thus, some level of extrapolation is required for interpretation of the functional results. However, abolition of firing seems unlikely to be due to effects on the intrafusal muscle fibres, but rather on some aspect of receptor potential generation, its modulation or its encoding. The present study, by its nature, could not determine precise loci or mechanisms of action for all of the functional aspects explored. Nevertheless, it is clear that calcium plays a range of very powerful and significant roles, at different locations, to affect muscle spindle firing. Moreover, it is hoped that these observations will identify the types of calcium‐dependent processes that might be explored more fully in future, more focussed studies.

### 4.1 Calcium buffering and known calcium‐dependent processes

The presence of at least six different calcium‐buffering proteins, which importantly include parvalbumin, often simply regarded as a peripheral mechanosensory neuron marker, indicates that regulation of intracellular calcium levels is of vital importance. One function likely to require calcium buffering in the sensory terminals is SLV recycling. Although spindle sensory terminals are not synaptic, their SLVs are part of an autogenic sensory regulatory system. Given this situation, another factor that must be borne in mind when interpreting the outcomes of the present experiments is that blocking calcium influx may stop SLV exocytosis of glutamate, thereby preventing activation of the terminal glutamate receptor (GluK2; Thompson et al., 2024). Blocking this receptor can entirely silence the stretch‐evoked response (Bewick et al., 2005). Such effects occur over tens of minutes to several hours, meaning it is important to also consider the time course of any effects. It should also be noted that none of the drugs seems to have any dramatic effect on spontaneous (prestretch) firing.

#### 4.1.1 P/Q‐type calcium channels

Two different VGCCs were found to regulate muscle spindle stretch‐evoked firing: Ca_v_2.1 (P/Q type) and Ca_v_1.1–1.4 (L‐type) HVA channels. Blocking Ca_v_2.1 (P/Q type) or Ca_v_1.1–1.4 (L‐type) HVA channels both increased hold‐phase firing, but with different patterns. Although it was not possible to differentiate between P‐type Ca_v_2.1 and Q‐type Ca_v_2.1 channels with the high ω‐agatoxin‐IVA concentration used (Catterall et al., 2005; Mintz et al., 1992; Sather et al., 1993) to overcome the access barrier of the spindle capsule, Ca_v_2.1 blockade clearly produced the largest (3‐fold) increase in hold‐phase firing. This would prevent synaptic release from the fusimotor or extrafusal neuromuscular junctions, indicating actions here to activate intrafusal muscle fibres are unlikely to be responsible. Conversely, Ca_v_2.1 blockade did not change FM1‐43 labelling, indicating its action was not due to enhancing SLV recycling, and indeed that it is not the source of the calcium regulating SLV recycling. Finally, it is clearly not contributing to the receptor potential directly, since channel blockade enhances, rather than inhibits, firing. Overall, therefore, these data indicate Ca_v_2.1 plays a negative feedback regulatory role.

#### 4.1.2 L‐type calcium channels

The observations in the present study confirmed that Ca_v_1.1–1.4 channels (L‐type) are also important for normal spindle functionality (Fischer & Schafer, 2002). In their very comprehensive study of the effects of nifedipine (5–25 µM) on isolated spindles from cat tenuissimus muscles, Fischer & Schafer (2002) also observed at higher concentrations an initial transient increase in firing, followed by a slow‐onset and prolonged reduction of firing. The excitation effect was maximum at 10 µM, with higher concentrations producing inhibition, essentially identical to our observations. Given that L‐type calcium channels also trigger calcium release from intracellular stores in skeletal muscle fibres, including the spindle intrafusal fibres, Fischer and Schäfer studied effects on intrafusal fibre tension, both by tension recording and visual observation. It was concluded that the excitatory action of nifedipine at these low concentrations was due to increased stiffness of the intrafusal fibres at the spindle poles, which would more faithfully transmit the length increase to the sensory endings. However, the inhibitory action could not be explained by changing mechanical properties of the intrafusal fibres, since inhibition often paralleled the initially predominant excitatory mechanical effects. Fischer and Schäfer used isolated spindles, and therefore concluded inhibition of firing at higher concentrations must be due to a hyperpolarisation action on the sensory terminals themselves. They also reasoned that the strong inhibitory effect at higher nifedipine concentrations suggests these calcium channels are open during the resting state, implying the sensory ending membrane potential is highly dependent on a sustained calcium conductance through L‐type channels. While this would correlate with the widespread distribution of SK2 K_Ca_s present in spindle afferent terminals (Shenton et al., 2014) and the requirement for strong calcium buffering indicated by the multiple buffering proteins, it could explain the excitation at low nifedipine concentrations, closing the SK2 channels, and hence depolarising the terminals. Dihydropyridines also block acetylcholine receptors, which are expressed by intrafusal fibres at contact areas with spindle sensory terminals (López et al., 1993). These receptors are themselves inhibitory (Gerwin et al., 2019) so blocking their inhibitory action could also contribute to the increased firing seen.

The mechanism of the later inhibitory action of nifedipine is less clear, but some clues may come from considering the increased FM1‐43 labelling with 10 µM nifedipine. First, this indicates L‐type channels can regulate SLV recycling, although which aspect (the uptake into [endocytosis] or release from [exocytosis] the terminals) is unclear initially. Our previous experiments show blocking all calcium channels with cobalt entirely blocks labelling of the sensory terminals (Bewick et al., 2005). If we assume the low level of labelling in normal calcium control experiments represents a net of recycling (endocytosis – exocytosis), and hence some dye is lost before imaging, then brighter labelling in the presence of nifedipine would indicate inhibition of this dye release. This, in turn, would inhibit glutamate release, and in the longer term reduce the sensitivity of the terminal to stretch, and inhibit stretch‐evoked firing. Thus, this would indicate L‐type channels may support exocytosis.

In contrast to the complexities of responses to nifedipine, taicatoxin produced a much simpler outcome of sustained increase of hold‐phase firing at any concentration. The increase exceeded that of nifedipine. Given the foregoing discussion, and that taicatoxin is also an SK channel antagonist, the even higher excitation with this toxin could result from the additive effects of increased stiffness of the intrafusal fibres at the spindle poles plus sensory terminal depolarisation from the block of SK channels present in the terminals and the preterminal axon. The toxin's rather greater potency at SK channels (1–10 nM) than at L‐type channels (10–500 nM) suggests the latter action at SK channels is likely to predominate. This seems to be the case even at 300 nM, as no corresponding inhibition is seen, unlike high concentrations of nifedipine. Overall, therefore, the evidence suggests L‐type calcium channels are expressed on the sensory terminals themselves, selectively regulating SLV exocytosis and the SK channel‐induced hyperpolarisation. However, this hypothesis clearly needs further study.

#### 4.1.3 N‐type calcium channels

Among the other HVA VGCCs, although Ca_v_2.2 (N‐type) channels contribute to synaptic release of primary afferent synaptic terminals in the superficial layers of the spinal cord (Westenbroek et al., 1998), are present in dorsal root ganglia (Chi et al., 2009) and contribute to nociception (Just et al., 2001), we found no evidence for them contributing to spindle mechanosensory responsiveness.

#### 4.1.4 Calcium‐activated potassium channels

Macromolecular complexes of VGCCs and K_Ca_s ensure signals from excitable cells are precisely localised in time and space (Berkefeld et al., 2006). The complex integration processes in neurons requires substantial channel diversity. Consequently, different members of both channel families are often co‐expressed to create regulatory macrodomains (Edgerton & Reinhart, 2003; Marrion & Tavalin, 1998; Prakriya & Lingle, 1999), including direct coupling via cytoskeletal proteins (Lu et al., 2007).

Blockers for any of the K_Ca_s increased firing, consistent with K_Ca_1.1 (BK), K_Ca_2.1–2.3 (SK) and K_Ca_3.1 (IK) channels regulating afferent discharge rates. Our own studies show that K_Ca_s, and in particular SK2.2 but not any other SK2 channel, are expressed both in muscle spindle sensory terminals and in the preterminal axon (Shenton et al., 2014). The location of the K_Ca_1.1 (BK) and K_Ca_3.1 (IK) channels is unknown. Our evidence here suggests channels of all K_Ca_ classes are expressed and functionally important. Toxins targeting BK/K_Ca_1.1 (iberiotoxin, antagonist, reversible increase; NS1619, irreversible potent/specific agonist, irreversible total inhibition) or IK/K_Ca_2.3 (TRAM‐34, antagonist, reversible increase) had strong effects on firing. Blocking both (charybdotoxin) produced the greatest enhancement of firing, relative to blocking either alone. Similarly, SK/K_Ca_1.1–1.3 antagonism (apamin, taicatoxin) also increased firing somewhat more modestly. Typically, the smaller increases in firing provoked by SK blockers could indicate they simply carry smaller conductances, or are located further away from the discharge encoding site, or some combination of the two. Calcium entry through Ca_v_2.1 is known to activate K_Ca_s, particularly K_Ca_1.1 (BK), triggering potassium efflux, thereby hyperpolarising and reducing firing in excitable cells (Edgerton & Reinhart, 2003; Fakler & Adelman, 2008; Piskorowski et al., 2008). Conversely, blocking calcium entry would depolarise and explain the observed increased firing frequency. There could also be an association with different classes of HVA VGCCs, particularly Ca_v_1.1–1.4 (L‐type) with SK2.2 in the terminal, and Ca_v_2.1 with K_Ca_1.1 (BK) and K_Ca_3.1 (IK) at the encoding site, giving much stronger control, and correlating with their much stronger effects on firing. Therefore, HVA VGCCs and K_Ca_s seem to serve as negative feedback regulators of spindle terminal excitability, as reported in many other systems, including firing rates (Faber & Sah, 2003) neurotransmission (Robitaille & Charlton, 1992), endocrine secretion (Findlay et al., 1985), auditory hair cell responses (Mammano et al., 2007), vascular (Ledoux et al., 2006), urinary bladder (Petkov & Nelson, 2005) and respiratory muscle tone (Kotlikoff, 1993).

### 4.2 TRP channels

Another goal for this study was to test if TRP channels might conduct the stretch‐sensitive calcium current of the receptor potential, and serve a potential role in SLV recycling. We found strong evidence that TRP channels, particularly the most widely posited mechanosensory TRP channel TRPV4 (Zhang et al., 2023), is functionally very important. Selective antagonists entirely blocked firing, while a potent selective agonist opposed this effect. Thus, TRPV4 seems to be expressed and essential for maintaining stretch‐evoked firing, further underlining the essential role of calcium. It is particularly striking that while antagonism entirely ablated firing, agonism increased it only marginally. Since further agonism has little additional effect on firing, this suggests TRPV4 channels are normally maximally open. What is less clear is whether TRPV4 is simply open all of the time, regardless of stretch, or whether it is opened by the stretch, and thus is a candidate for the stretch‐sensitive calcium current of the receptor potential (Hunt et al., 1978). Given the previous observations with other channels, TRPV4 is certainly the prime candidate for this role.

What is also unclear is how TRPV4 so powerfully regulates the stretch‐evoked response of the muscle spindle afferents. Since removal of external calcium, in the continued presence of sodium, has no effect on the receptor potential (Hunt et al., 1978), it seems unlikely that TRPV4 blockade simply reduces the receptor potential. Therefore, its action seems to be downstream of the depolarisation itself. Rather, this calcium entry could be to enable the calcium‐dependent glutamate secretion from SLVs that maintains the ending stretch‐sensitivity. The increased retention of FM1‐43 with TRPV4 block would support this view. Therefore, antagonists would prevent secretion, and the ending would lose the ability to respond to stretch (Bewick et al., 2005). This would be in agreement with other studies, since TRPV4 channels regulate the exocytosis of secretory granules from Merkel cells (Boulais et al., 2009). One final consideration is whether blockade of Piezo2, a non‐selective cation channel (Coste et al., 2010), might play a role in any of the observed inhibitory effects within the selectivity range of the drugs used. While there is evidence Piezo1 interacts with TRPV4 (Servin‐Vences et al., 2017), we are not aware of any evidence for Piezo1 in muscle spindle primary afferent endings, nor for any of the ligands we have studied blocking or inhibiting Piezo2 directly. Thus, from first principles, it seems most parsimonious currently to limit our interpretations as attributable to the channels considered here.

### 4.3 Summary

**FIGURE 6:**
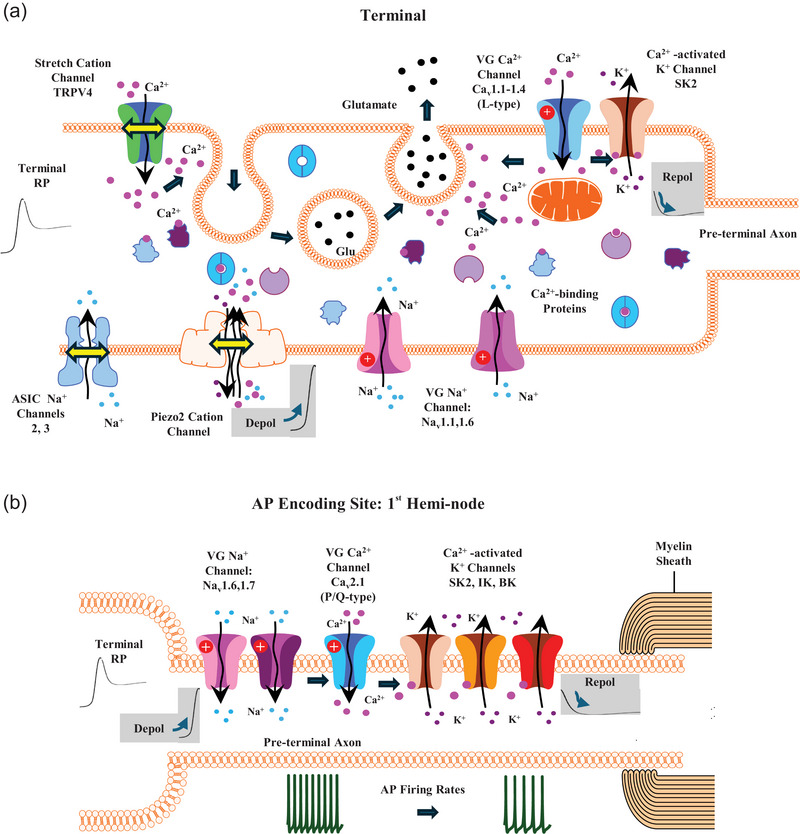
Summary diagram of proposed calcium signalling, relative to other key channels currently thought to be acting in mechanosensory terminals. (a) Stretch induces a depolarising receptor potential (RP) involving the opening of one or more of the following: Piezo2 (Woo, et al., 2015), ASIC2 (Bornstein, et al., 2024), ASIC3 (Lin, et al., 2016) and, probably indirectly, voltage‐gated sodium channels Na_v_1.1 or Na_v_1.6 (Carrasco, et al., 2017). The present study indicates these stimuli (stretch, depolarisation) induce the following calcium signalling events. Stretch opens TRPV4, for a calcium influx to enable SLV endocytosis. The depolarisation opens L‐type (Ca_v_1.1–1.4) voltage‐gated calcium channels from which calcium triggers SLV exocytosis. In addition, the calcium opens SK2 (K_Ca_2.2) potassium channels. Given this sequential nature, and the calcium diffusion time, the resulting partial repolarisation of the terminal would be delayed relative to its initial peak dynamic depolarisation. The L‐type channel long open time, and the resulting prolonged calcium influx, together with substantial intracellular calcium buffering to prevent calcium‐dependent channel inactivation (Snutch, et al., 2005), could extend SK2 open time to maintain the repolarisation throughout a sustained stretch. This complex depolarisation wave spreads electrotonically, or perhaps even propagates as an AP (Carrasco, et al., 2017) (Housley, et al., 2024), to the preterminal axon. (b) The RP‐induced depolarisation of the preterminal axon opens voltage‐gated sodium channels (Na_v_1.6 and 1.7, Carrasco, et al., 2017), initiating a very high frequency AP burst typical of a dynamic stretch‐onset response. The present study indicates these events then, sequentially, gate P/Q‐type (Ca_v_2.1) calcium channels. The incoming calcium opens further calcium‐activated potassium channels (SK2, K_Ca_2.2; IK, K_Ca_3.1; BK, K_Ca_1.1), repolarising the preterminal axon, reducing the afferent discharge firing rate to a sustained level until the terminal length decreases upon release. Created using Motifolio.

These multiple calcium signals presumably underlie, at least in large part, the complexity and control of the generation of the receptor potential (RP) and its encoding in the afferent output, and are summarised in Figure 6. The current generating the RP has sodium, potassium and calcium components, with some of the potassium components being TEA sensitive and others not, and the calcium component being stretch‐evoked (not voltage‐gated) and only revealed if sodium is absent. Some phases of the RP are particularly sensitive to the rate of stretch, which then at least partially and very rapidly turn off on reaching a steady length. A residual component then reports the sustained length, and final components return the terminal potential to its resting state on release. The various RP components are faithfully encoded into AP frequency at encoding sites thought to correspond with the heminodes of the Ia afferent branches supplying the separate terminals comprising the complete primary ending. These transductions, potentials and encodings all have varying kinetics, from the order of milliseconds to seconds. In addition, there is the SLV‐secreted glutamate acting over the timescale of tens of minutes to hours. Together, the resultant output is remarkably adaptable, maintaining sensitivity over a large range of muscle lengths, yet simultaneously remarkably consistent between individual spindles and even between individual animals. We would propose that at least many of these complexities reflect the intricate control mechanisms resulting from the presence and actions of the calcium channels, calcium‐activated channels and their resulting signals reported here. SK2 in the terminals is a candidate to regulate the repolarisation of the RP on reaching maximal length, and then eventual release. Direct stretch, opening TRPV4, would be an ideal source for this calcium to activate SK2. Finally, SK2, expressed at the AP encoding site(s), together with BK and IK, seem likely to be to fine‐tune the local membrane potential, via the opening of voltage‐gated (P/Q‐type) calcium channels, to ensure faithful translation of the complex RP waveform into an appropriately modulated AP frequency, from very low frequencies up to 200–500 impulses per second. Then, over the longer‐term, to ensure that the terminals maintain their sensitivity to stretch, glutamate release, triggered by calcium entry through TRPV4 and L‐type channels, activates the autogenic glutamate receptor GluK2. Thus, none of these systems seems redundant, but rather each regulates specific components of this sequence. Of course, even this can only be a partial explanation, since how other powerful components such as Piezo2 might contribute, and how GluK2's activation of phospholipase D regulates terminal overall responsiveness, still remain to be elucidated. Finally, it seems likely it is this very complexity that enables animals to execute exquisite motor actions with great precision and accuracy on a very regular basis, from non‐human mammals hunting and catching prey to humans performing the remarkable feats of agility of Olympic gymnasts.

## AUTHOR CONTRIBUTIONS

All aspects of the work were performed in the laboratory of Guy Smith Bewick. All authors contributed to acquisition, analysis or interpretation of data for the work and drafting of the work or revising it critically for important intellectual content. Guy Smith Bewick, Robert William Banks, and Anna Simon also contributed to conception or design of the work. All authors have read and approved the final version of this manuscript and agree to be accountable for all aspects of the work in ensuring that questions related to the accuracy or integrity of any part of the work are appropriately investigated and resolved. All persons designated as authors qualify for authorship, and all those who qualify for authorship are listed.

## CONFLICT OF INTEREST

None declared.

## Data Availability

All data are available upon request from the corresponding author.
